# A Novel Cell-Cell Communication Structure: Tanycyte and Cilia Hypothalamic Unifying Glia-cilia Structure (HUGS)

**DOI:** 10.17912/micropub.biology.001961

**Published:** 2026-01-13

**Authors:** Kara R. Schwantz, Jaelyn G. Boone, Kathryn M. Brewer, Nicolas F. Berbari

**Affiliations:** 1 Biology, Indiana University Indianapolis, Indianapolis, Indiana, United States; 2 Department of Biology, Indiana University Indianapolis, Indianapolis, Indiana, United States

## Abstract

Primary cilia, microtubule-based sensory organelles that mediate cell–cell communication, may facilitate signaling in the brain through direct physical contacts (e.g., synapse-like structures). Similarly, specialized glial cells lining the third ventricle (3V) called tanycytes signal through physical interactions and can dynamically alter their morphology in response to external stimuli and physiological changes. Here, we identify robust cilia-tanycyte contacts; we term HUGS ( H ypothalamic, U nifying G lia-cilia S tructures) and discover that these connections are disrupted in a mouse ciliopathy model ( *Bbs4* ) exhibiting hypothalamic dysfunction. These data provide insight into potentially new cell-cell signaling mechanisms deployed by neuronal cilia.&nbsp;

**Figure 1. Defining and Assessing HUGS&nbsp;in the mouse hypothalamus &nbsp;:**
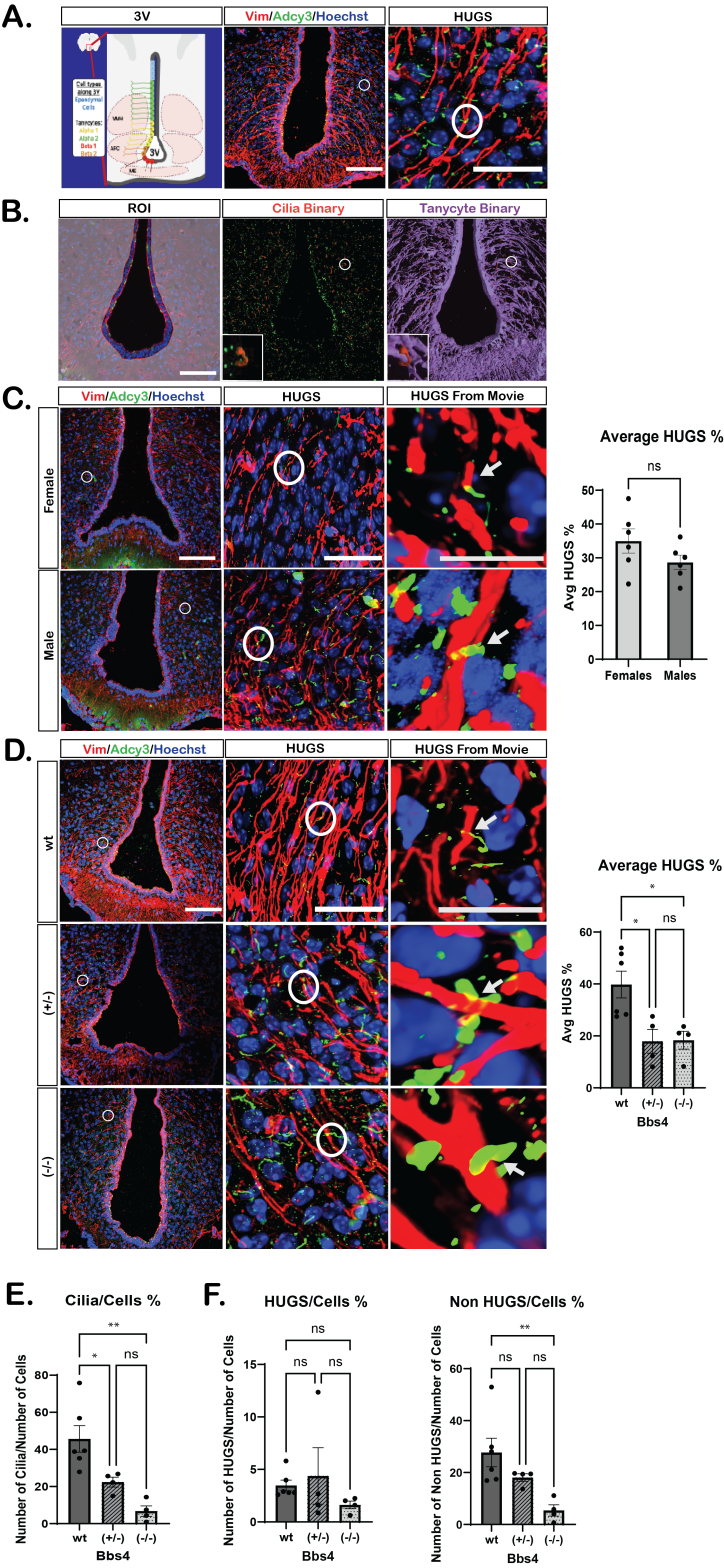
**A.** *(Left)* Schematic of the third ventricle (3V) of the mouse brain. Ependymal cells (blue) and tanycyte populations (Alpha 1: yellow, Alpha 2: green, Beta 1: red, Beta 2: orange). Neuroanatomical nuclei and structures of the hypothalamus are also indicated including the ventromedial hypothalamus (VMH), arcuate nucleus (ARC), and the median eminence (ME). *(Middle)* Confocal immunofluorescence image of the ventricle with tanycytes (Vim, red), cilia (Adcy3, green), and nuclei (Hoechst, blue). Scale bar 100µm. *(Right)* Inset from the same image with an example of cilia HUGS, indicated with a white circle. Scale bar 30µm. **B.** *Computer assisted HUGS analysis.* *(Left)* Region Of Interest (ROI, gray) is defined to exclude the 3V space. &nbsp; *(Middle)* Binaries for cilia (red) are defined using thresholding. Inset indicated with a white circle shows the binary (red) for individual cilia. *(Right)* Binaries for tanycytes (purple) are defined using thresholding. Inset indicated with a white circle shows the binary for cilia (red) and tanycytes (purple). **C.** *Assessing HUGS of wildtype adult male and female mice.* Confocal immunofluorescence image of tanycytes (Vim, red), cilia (Adcy3, green) and nuclei (Hoechst, blue). Scale bar 100µm. Inset of cilia HUGS indicated with a white circle. Scale bar 30µm. An individual HUGS structure from a 3D movie (HUGS From Movies 1 and 2). Scale Bar 20µm. Quantification of average number of HUGS (%) between female and male mice shows no statistical significance (One-Way ANOVA test). **D.** *Assessing HUGS of a mutant ciliopathy mouse model.* Confocal immunofluorescence image of tanycytes (Vim, red), cilia (Adcy3, green) and nuclei (Hoechst, blue) of *Bbs4* wildtype (wt), heterozygous (+/-) and mutant (-/-) animals. Scale bar 100µm. Inset of HUGS indicated with a white circle. An individual HUGS structure from a 3D movie (HUGS From Movies 3, 4 and 5).Scale bar 20µm. Quantification of average number of HUGS % between wt, +/-, and -/- male and female animals shows a significant reduction in HUGS of +/- and -/- compared to wt littermates (One-Way ANOVA test, * indicates p < 0.05). E. Quantification of the percentage of Adcy3+ cilia to number of cells in wt, +/-, and -/- animals. (One-way ANOVA, * indicates p<0.05, ** indicates p<0.0001). F. Quantification of the percentage of Adcy3+ cilia HUGS and Non-HUGS to number of cells in wt, +/-, and -/- animals. (One-way ANOVA, * indicates p<0.05, ** indicates p<0.0001).

## Description

Primary cilia are present on nearly all hypothalamic cell types including neurons (Bishop et al., 2007; Jurisch-Yaksi et al., 2024) and are well established as sensory organelles. Their functions in the CNS are best characterized in vision and olfaction, where specialized G protein–coupled receptors localize to the ciliary membrane, detect extracellular ligands, and initiate signaling cascades essential for sensory perception (Singla & Reiter, 2006). In hypothalamic neurons, primary cilia similarly regulate feeding and circadian rhythms through ligand–receptor signaling (Davenport et al., 2007; Ojeda-Naharros et al., 2025; Oya et al., 2024; Xun et al., 2025) (Bernard et al., 2023; Siljee et al., 2018; Tu et al., 2023; Wang et al., 2021). Recent evidence further suggests that neuronal cilia may not only receive external cues but also engage in direct, contact-dependent communication with neighboring cells, in some cases resembling synapse-like interactions (Ott et al., 2024; Sheu et al., 2022; Volos et al., 2025; Wu et al., 2024). These expanding roles for ciliary signaling raise important questions about how sensory information from outside the brain is relayed to hypothalamic circuits that regulate physiology.

Tanycytes are specialized, highly polarized glial cells lining the third ventricle and represent a compelling candidate for mediating this communication. Unlike typical ependymal cells, tanycytes consist of distinct subtypes (α1, α2, β1, β2) and display pronounced apical–basal polarity (Dali et al., 2023). Their apical surfaces extend primary cilia into the cerebrospinal fluid, while their elongated basal processes project deep into hypothalamic parenchyma, where they contact neurons, glia, and blood-vessel pericytes (Mullier et al., 2010; Pasquettaz et al., 2021). This architecture enables tanycytes to integrate peripheral signals by passively sensing molecules such as glucose and actively transporting hormones and neuropeptides like insulin and leptin into the brain in order to influence neuronal pathways regulating energy homeostasis (Balland et al., 2014; Frayling et al., 2011; Porniece Kumar et al., 2021). Together, these features position tanycytes as a potential interface through which peripheral metabolic cues could engage neuronal ciliary signaling to shape hypothalamic function.

Building on this idea, we asked whether tanycyte processes might physically engage neuronal cilia to provide input into hypothalamic circuits. To test this, we immunostained tanycytic processes with vimentin and labeled neuronal cilia with adenylate cyclase III (Adcy3) (Bishop et al., 2007; Robins et al., 2013; Schnitzer et al., 1981). We hypothesize that tanycytic processes form specialized structural contacts with neuronal cilia in the hypothalamus, in a manner similar to the known ability of cilia to eavesdrop on synapses in the human cortex, differentiated hypothalamic neurons and mouse hippocampus (Wu et al., 2024).

Using confocal fluorescence microscopy, we developed an assay to identify and analyze these tanycyte–cilia contacts which we term H ypothalamic U nifying G lia–cilia S tructures or HUGS for short ( **Figure 1A** ). We used a computer-assisted image analysis pipeline adapted from our previously reported cilia analysis methods using NIS Elements (Brewer et al., 2024; Brewer et al., 2023). Within defined regions of interest (ROI), we generated binary masks for cilia (cilia binary) and tanycytic processes (tanycyte binary) separately ( **Figure 1B** ). We then established a “parent–child” hierarchical structure in which cilia binaries were assigned as children to the closest parent, i.e. tanycyte binary. The distance between the two structures was calculated, and contacts with a measured distance of 0μm were classified as HUGS, indicating potentially direct contact between a cilium and a tanycytic process. This rapid, unbiased approach enabled high-throughput quantification of hundreds of HUGS across multiple images per animal, yielding a comprehensive view of their frequency and characteristics while increasing our sensitivity to detect subtle changes.

We first applied this approach in adult C57Bl/6 male and female mice. Although the hypothalamus is a sexually dimorphic brain region, including tanycytes themselves (Ciofi et al., 2006; Prevot et al., 2018; Simerly et al., 1997), we detected no sex-specific differences in the number of HUGS, with ~30% of cilia within the ROIs forming HUGS with tanycytic processes ( **Figure 1C** and **Movies 1** and **2** ).

We next asked whether HUGS are altered in a ciliopathy model that is known to be associated with hypothalamic dysfunction. Using Bardet–Biedl syndrome 4 gene ( *Bbs4* ) mutant mice, we observed a significant reduction in HUGS in both heterozygous ( *Bbs4 ^+/−^* ) and homozygous ( *Bbs4 ^−/−^* ) mutants compared to wildtype sibling controls ( **Figure 1D** ). Interestingly, it is well-characterized that neuronal cilia marker *Adcy3* is less frequent in adult ciliopathy mouse brains which we also observe in our samples ( **Figure 1E** ) (Agassandian et al., 2016; Agassandian et al., 2014; Berbari et al., 2008). To address if the reduction in HUGS of mutant animals was correlated with an overall reduction in cilia abundance or total cell number rather than cilia-tanycyte interactions, we normalized the number of HUGS to total cell count using a threshold for Hoechst staining. This allowed us to quantify and assess total cilia, which includes those engaged in HUGS and all other cilia compared to the total number of cells within each ROI ( **Figure 1F** ). While HUGS per total cell count were not significantly different across genotypes, total cilia were reduced in a genotype–dependent manner with wildtype (wt) animals having more than heterozygotes ( *Bbs4 ^+/−^* ) which had more than mutants ( *Bbs4 ^−/−^* ) ( **Figure 1E** and **Movie 3, 4** and **5** ). These data demonstrate that tanycytes and cilia interact in the hypothalamic parenchyma and that these interactions are altered in a ciliopathy model.

Together, these findings demonstrate that tanycytes potentially form direct physical contacts with neuronal primary cilia in the mouse hypothalamus. By establishing a quantitative framework for analyzing these interactions, we show that HUGS are a robust and consistent feature across sexes but are disrupted in a ciliopathy model. Given their location, HUGS may relay metabolic or hormonal cues from the cerebral spinal fluid or serum directly onto ciliated neurons in the hypothalamus, modulating their activity. Additionally, HUGS may serve to stabilize cilia structure or regulate access of receptors and adhesion molecules at the ciliary membrane. These ideas all suggest that tanycyte–cilia contacts (HUGS) represent an underappreciated form of glia–neuron communication with potential relevance for hypothalamic signaling in health and disease.

Having established this framework, our next steps are to expand analyses of HUGS in ciliopathy mice by incorporating measures such as the ratio of HUGS to total cilia. We also plan to investigate how HUGS prevalence and structure vary across developmental stages and aging, and to determine whether HUGS preferentially form with specific neuronal or glial subtypes or in association with distinct ciliary receptors and potentially deploy specific adhesion molecules recently identified in cilia proteomics approaches in the brain (Chang et al., 2025). These studies will clarify whether HUGS represents a general principle of hypothalamic organization or a specialized signaling mechanism with cell-type and receptor specificity.

## Methods

*Mouse Lines*

Mice were housed under a standard 12-hour light/dark cycle with *ad libitum* food and water. All animal protocols and procedures were performed in accordance with the Institutional Animal Care and Use Committee (IACUC) at Indiana University - Indianapolis. Adult (8-week-old) male and female C57Bl/6J (stock #000664) and Bbs4 (B6.129- *Bbs4 ^tm1Vcs^* /J Stock #010728) mice were used for analyses (Mykytyn et al., 2004).

*Tissue Collection*

Tissue was collected at 8 weeks of age, previously described (Brewer et al., 2024). Briefly, the mice were anesthetized with 0.1 mL/10 g of body weight dose of 2.0% tribromoethanol (Sigma Aldrich, St. Louis, MO, USA) and perfused transcardially with PBS, followed by 4% paraformaldehyde (PFA) (catalog no. 15710, Electron Microscopy Sciences, Hatfield, PA, USA). Brains were isolated and postfixed in 4% PFA for 4 hours at 4 °C and then cryoprotected with 30% sucrose in PBS for 16–24 hours at 4 °C. Brains were embedded in optimal cutting temperature compound (OCT) and cryosectioned at 15 µm directly onto slides for staining.

*Immunofluorescence&nbsp;*

Cryosections were washed twice with PBS for 5 min and then permeabilized and blocked in a PBS solution&nbsp;containing&nbsp;1% BSA, 0.3% Triton X-100, 2% (v/v) donkey serum, and 0.02% sodium&nbsp;azide&nbsp;for&nbsp;30&nbsp;min at&nbsp;RT. The sections were incubated with primary antibodies at 4 °C.&nbsp;The primary antibodies included cilia marker&nbsp;Adcy3&nbsp;(1:1000 dilution; catalog no. CPCA-ACIII,&nbsp;EnCor, Gainesville, FL, USA) and&nbsp;Vimentin (1:300 dilution;&nbsp;catalog no. EPR3776,&nbsp;Abcam,&nbsp;Waltham, MA, USA). The sections were then washed&nbsp;twice&nbsp;for 5 min&nbsp;with PBS&nbsp;and&nbsp;3&nbsp;times&nbsp;for 5 min&nbsp;with blocking solution&nbsp;described above. Then sections were&nbsp;incubated&nbsp;in&nbsp;secondary antibodies for&nbsp;1.5 hours&nbsp;at room temperature. The secondary antibodies include donkey conjugated Alexa Fluor&nbsp;488, and 647 (1:1000;&nbsp;Invitrogen, Carlsbad, CA, USA&nbsp;and Jackson&nbsp;Immuno&nbsp;Research,&nbsp;West Grove, PA,&nbsp;USA)&nbsp;against&nbsp;appropriate&nbsp;species&nbsp;according to the corresponding primary.&nbsp;&nbsp;The slides were then washed in PBS and counterstained with Hoechst nuclear stain (1:1000; catalog no.&nbsp;H3570,&nbsp;Thermo&nbsp;Fisher Scientific) for 5 min at room temperature. Coverslips were mounted&nbsp;using&nbsp;SlowFade&nbsp;Diamond Antifade&nbsp;Mountant&nbsp;(catalog no. S36972,&nbsp;Thermo&nbsp;Fisher Scientific).&nbsp;All primary and secondary solutions were made in the blocking solution described above.&nbsp;

*Imaging and Analysis*

Images were captured using a Nikon Ax confocal microscope (40X water lens objective) and cilia HUGS were identified and analyzed using Nikon Elements Software (NIS Elements). Computer-assisted cilia analysis was performed as previously described (Bansal et al., 2021; Brewer et al., 2024; Brewer et al., 2023). Thresholding was used to identify cilia, tanycytes, and Hoechst-positive nuclei. As part of our approach, cilia were identified to be objects &nbsp; 1.5 μm in length. Any background staining was eliminated through exclusion filtering of the cilia threshold binary by sphericity &nbsp;&nbsp; 0.850 (value 1 = perfect sphere) and large volume staining ≥60.0 μm ^3^ . &nbsp;Once parents (tanycyte binaries) and children (cilia binaries) were identified, distances between the child and the closest parent were measured. Distances of 0 μm between a parent and child were counted as HUGS.

## Reagents

**Table:** 

***Reagent*** *&nbsp;*	***Company (Cat.&nbsp;No.)*** *&nbsp;*	***Application*** *&nbsp;*
*Vimentin&nbsp;antibody&nbsp;*	*Abcam&nbsp;(EPR3776)&nbsp;*	*Tanycyte&nbsp;marker&nbsp;*
*ACIII&nbsp;antibody&nbsp;*	*EnCor&nbsp;(CPCA-ACIII)&nbsp;*	*Cilia marker&nbsp;*
*Hoechst&nbsp;*	*ThermoFisher&nbsp;(H3570)&nbsp;*	*Nuclei stain&nbsp;*
*Donkey&nbsp;Anti-Rabbit 488&nbsp;*	*Invitrogen&nbsp;(A31573)&nbsp;*	*Secondary antibody&nbsp;*
*Donkey&nbsp;Anti-Chicken 647&nbsp;*	*Jackson&nbsp;Immuno&nbsp;Research&nbsp;(703-605-155)&nbsp;*	*Secondary antibody&nbsp;*
*16%&nbsp;Paraformaldehyde&nbsp;*	*E.M.S.&nbsp;(15710)&nbsp;*	*Tissue fixation&nbsp;*
*Optimal Cutting Temperature Embedding Medium&nbsp;*	*Fisher&nbsp;(4585)&nbsp;*	*To&nbsp;cryoprotect&nbsp;tissue specimens&nbsp;*
*ProLong Diamond Slow Fade&nbsp;Mountant&nbsp;&nbsp;*	*ThermoFisher&nbsp;(P36970)&nbsp;*	*Mount slides after staining&nbsp;*

## Data Availability

Description: Movie 1 Male HUGS which corresponds to data in third column of 1C. Resource Type: Audiovisual. DOI: https://doi.org/10.22002/n6zc5-6q846 Description: Movie 2 Female HUGS which corresponds to data in third column of 1C. Resource Type: Audiovisual. DOI: https://doi.org/10.22002/hsxaz-g6y86 Description: Movie 3 Bbs4 wildtype HUGS which corresponds to data in third column of 1D. Resource Type: Audiovisual. DOI: https://doi.org/10.22002/m6zjh-d0390 Description: Movie 4 Bbs4 heterozygote HUGS which corresponds to data in third column of 1D. Resource Type: Audiovisual. DOI: https://doi.org/10.22002/30vph-fn897 Description: Movie 5 Bbs4 mutant HUGS which corresponds to data in third column of 1D. Resource Type: Audiovisual. DOI: https://doi.org/10.22002/5n2jk-man67
